# The integration of single-cell sequencing, TCGA, and GEO data analysis revealed that PRRT3-AS1 is a biomarker and therapeutic target of SKCM

**DOI:** 10.3389/fimmu.2022.919145

**Published:** 2022-09-23

**Authors:** Wancong Zhang, Xuqi Xie, Zijian Huang, Xiaoping Zhong, Yang Liu, Kit-Leong Cheong, Jianda Zhou, Shijie Tang

**Affiliations:** ^1^ Department of Plastic Surgery and Burn Center, Second Affiliated Hospital, Shantou University Medical College, Shantou, China; ^2^ Plastic Surgery Institute of Shantou University Medical College, Shantou, China; ^3^ Guangdong Provincial Key Laboratory of Marine Biotechnology, Department of Biology, College of Science, Shantou University, Shantou, China; ^4^ Department of Plastic and Reconstructive Surgery, Central South University Third Xiangya Hospital, Changsha, China

**Keywords:** scRNA-seq, SKCM, PRRT3-AS1, lncRNAs, biomarker, TCGA, GEO

## Abstract

**Introduction:**

Skin cutaneous melanoma (SKCM) is the world’s fourth deadliest cancer, and advanced SKCM leads to a poor prognosis. Novel biomarkers for SKCM diagnosis and prognosis are urgently needed. Long non-coding RNAs (lncRNAs) provide various biological functions and have been proved to play a significant role in tumor progression. Single-cell RNA sequencing (scRNA-seq) enables genome analysis at the single-cell level. This study explored prognostic lncRNAs in SKCM based on scRNA-seq and bulk RNA sequencing data.

**Materials and methods:**

The TCGA cohort and melanoma samples in the GEO database (GSE72056, GSE19234, GSE15605, GSE7553, and GSE81383) were included in this study. Marker genes were filtered, and ensemble lncRNAs were annotated. The clinical significance of selected lncRNAs was verified through TCGA and GEO dataset analysis. SiRNA transfection, wound−healing and transwell assays were performed to evaluate the effect of PRRT3-AS1 on cellular function. Immune infiltration of the selected lncRNAs was also exhibited.

**Results:**

A 5-marker-lncRNAs model of significant prognostic value was constructed based on GSE72056 and the TCGA cohort. PRRT3-AS1 combined with DANCR was then found to provide significant prognostic value in SKCM. PRRT3-AS1 was filtered for its higher expression in more advanced melanoma and significant prognosis value. Cellular function experiments *in vitro* revealed that PRRT3-AS1 may be required for cancer cell migration in SKCM. PRRT3-AS1 was found to be related to epithelial-mesenchymal transition (EMT) signaling pathways. DNA methylation of PRRT3-AS1 was negatively related to PRRT3-AS1 expression and showed significant prognosis value. In addition, PRRT3-AS1 may suppress immune infiltration and be involved in immunotherapy resistance.

**Conclusion:**

PRRT3-AS1 may be a diagnostic and prognostic biomarker of SKCM.

## Introduction

Skin cutaneous melanoma (SKCM) is one of the most fatal human diseases and the fourth leading cause of cancer-related mortality worldwide (1). SKCM ranges from a benign neoplasm to a primary malignant neoplasm, for which the 5-year survival exceeds 95%, to a metastatic SKCM, for which the length of survival barely exceeds 1 year (1–3). The first choice for treating patients with primary SKCM is surgical resection, whereas advanced SKCM is highly aggressive, making radiotherapy and chemotherapy necessary and early detection significant (4). Although markers like BRAF and NRAS mutation and immune checkpoints like PD-L1 have greatly contributed to the accurate diagnosis and prognosis of SKCM patients (5, 6), challenges still exist in exploring other specific molecular characteristics to improve the prognosis and treatment of advanced SKCM (6).

RNAs can be divided into protein-coding messenger RNAs and functional noncoding RNAs, the latter of which was once considered to be coded by “junk” DNAs. Recently, various functional noncoding RNAs, such as small nuclear RNAs and longer noncoding transcripts, have been discovered and described (7). Long noncoding RNAs (lncRNAs), usually defined as non-protein-coding transcripts longer than 200 nt, are involved in diverse functions, such as epigenetic regulation of allelic expression (8) and developmental differentiation (9). LncRNAs can modulate gene expression through mechanisms including chromatin modification, transcriptional activation, RNA editing/splicing/degradation, and translational efficiency regulation (10). Genome-wide association studies (GWAS) of tumor samples have identified numerous lncRNAs associated with various types of cancer (11). In SKCM, several lncRNAs were differentially expressed and acted as potential regulators of tumor progression and metastasis (12). However, current knowledge on the downregulation and mechanisms of lncRNAs in SKCM is far from complete (12).

ScRNA-seq enables genome-wide gene expression analysis with single-cell resolution, providing unprecedented capabilities in the identification of cellular heterogeneity, the transition of cellular states, and intercellular communications in complex tissue (12). Therefore, a library constructed based on ployA tail enrichment could enable many lncRNAs to be discovered. Based on the GEO dataset and TCGA cohort including scRNA-seq data combined with Lasso regression analysis, we discovered a key lncRNA, PRRT3-AS1, which is an independent marker of SKCM metastasis. Further research suggested PRRT3-AS1 is a new diagnostic and prognostic marker for SKCM, and it could be a treatment target for SKCM.

## Materials and methods

### Data acquisition and processing

A total of 472 SKCM patients in the TCGA database and numerous melanoma samples in the GEO database (**Supplemental Table 1**) were included in this study. Raw data of selected GEO databases (http://www.ncbi.nih.gov/geo) were downloaded as MINiML files. The extracted data were normalized by log2 transformation by the normalize quantiles function of the preprocessCore package in R software. Probes were converted to gene symbols according to the annotation information of the normalized data in correspond platforms. Marker genes were filtered from GSE72056, and ensemble lncRNAs were annotated. Selected lncRNAs were verified through TCGA and GEO dataset analysis. RNA-sequencing expression (level 3) profiles and corresponding clinical information were downloaded from the TCGA dataset(https://portal.gdc.com). Raw data of selected GEO databases (http://www.ncbi.nih.gov/geo) were downloaded as MINiML files. The extracted data were normalized by log2 transformation by the normalize quantiles function of the preprocessCore package in R software. Probes were converted to gene symbols according to the annotation information of the normalized data in correspond platforms. Key functions of the selected lncRNAs were validated through a series of assays against cell model *in vitro*. All the R packages in the study were implemented by R software version 4.0.3. Original data including metadata of datasets involved, main R code and survival analysis results were collated as supplementary document (**Supplemental document 1-11**).

### Construction of the prognostic model and overall survival prediction performance and immunoinfiltration analysis of the model

A total of 4645 single-cell RNA-seq data points from 19 patients were included in the study. Marker lncRNAs were filtered by the criteria of log2|FC| > 0.5, FDR < 0.001, and RNA expressed percent > 60% based on Tumor Immune Single-cell Hub (http://tisch.comp-genomics.org) (13, 14). Tumor Immune Single-cell Hub is a scRNA-seq database focusing on tumor microenvironment (TME) and provides detailed cell-type annotation at single-cell level, enabling exploration of TME among different cancer types. The least absolute shrinkage and selection operator (LASSO) regression algorithm was used for feature selection to construct a marker model which 10-fold cross-validation was used, and the R package ‘glmnet’ was used for the analysis. Marker genes in melanoma cells were analyzed based on GSE72056 single cell sequencing data from 19 patients. Then marker genes with logFC>0.5, more than 60% positive expression in tumor cells and P <0.001 were selected for subsequent IncRNAs annotation. The obtained 10 up-regulated marker IncRNAs were put into the TCGA_SKCM cohort data for LASSO regression analysis, so as to screen the characteristic variables and construct the prognostic model. Clinical data was used to illustrate the prognostic value of the model. Log-rank test was used to compare differences in survival between these groups. Kaplan-Meier curves, p-values and hazard ratio (HR) with 95% confidence interval (CI) were generated by log-rank tests and univariate cox proportional hazards regression. Package GGRisk, survival and SurvMiner were applicated in the process. TimeROC analysis was used to compare the predictive accuracy of lncRNAs. *P *< 0.05 was considered to be statistically significant.

### Screening and validation of the prognostic lncRNAs from the established prognostic model

Multivariate and univariate Cox proportional hazard regression of risk factors were performed to identify the proper terms to build the nomogram. The forest was used to show the Pvalue, HR and 95% CI of each variable through ‘forestplot’ R package. A nomogram was developed based on the results of multivariate cox proportional hazards analysis to predict the 1, 5, and 10-year overall recurrence based on the TCGA-SKCM cohort. The nomogram provided a graphical representation of the factors associated with the prognostic model through ‘rms’ R package. To further verify the predictive performance of the two most promising lncRNAs, we also performed survival analysis in the Genomics Analysis and Visualization Platform (https://hgserver1.amc.nl/cgi-bin/r2/main.cgi) based on the GSE19234 dataset. Genomics Analysis and Visualization Platform is an online datamining and discovery platform designed to perform datascience tasks in the omics field.

### Differential expression validation of the filtered lncRNAs in other datasets

The Gene Expression Profiling Interactive Analysis 2 (GEPIA2) online tool (http://gepia2.cancer-pku.cn/#index) (15) was utilized to calculate PRRT3-AS1 and DANCR expression levels in the TCGA-SKCM cohort. GEPIA2 is a tool for analyzing the RNA sequencing expression data of 9,736 tumors and 8,587 normal samples from the TCGA and the GTEx projects, using a standard processing pipeline and provides customizable functions based on the datasets. GSE15605 (melanoma progression grouped expression profiling by array, 74 samples) and GSE7553 (malignant transformation and progression of metastatic melanoma grouped expression profiling by array, 87 samples) datasets were downloaded from the GEO database (https://www.ncbi.nlm.nih.gov/gds). Copy number variation analysis of PRRT3-AS1 was conducted using the cBioPortal online tool (https://www.cbioportal.org/) (16). The cBio Cancer Genomics Portal is an open-access resource for interactive exploration of multidimensional cancer genomics data sets.

### Cell culture and siRNA transfection

Two melanoma cell lines (A2058 and SK-MEL-28) were obtained from ATCC (American Type Culture Collection) and cultured in Dulbecco’s Modified Eagle Medium (DMEM; Gibco, Beijing, China) supplemented with 10% fetal bovine serum (FBS; Gibco, Australia) at 37°C in a 5% CO_2_ humidified incubator. Cells were plated into a 6-well plate at a density of 70–90%. The PRRT3-AS1 si-RNA (si- PRRT3-AS1) and its corresponding scrambled siRNA control (si-NC) were obtained from Miao Ling (China) (**Supplemental Table 2**). The siRNAs were separately transfected into cells using a Lipofectamine RNA iMAX reagent (Invitrogen, USA).

### Cell migration by wound−healing assay

A wound healing assay was performed to evaluate cell migration ability. Cells were seeded overnight in 6-well plates at a density of 10^5^ cells per well in 2 mL medium. After being transfected with siRNA, a straight linear wound was made in each well by using a 1 mL pipette tip (KIRGRN, Shanghai, China). Then, the cells were carefully washed with PBS to remove cell debris and cultured in DMEM supplemented with 5% FBS. Finally, wound healing images were taken at 0, 24 and 36 h by using an inverted microscope (ZEISS, axio observer A1) with a 200x objective.

### Cell migration by transwell assay

The migration capacities of cells were determined by a transwell assay. A2058 and SK-MEL-28 cells at a density of 1 × 10^5^ cells/well were inoculated above the 8 μm chamber (Corning Inc., NY-Corning, USA) with a medium containing 200 μL serum-free medium and below the chamber with a medium containing 10% FBS of 500 μL for 24 h. Finally, five fields were randomly selected to capture images, and the number of cells passing through the chamber was counted.

### PRRT3-AS1 function annotation

The Cancer Single-cells State Atlas (CancerSEA) online database (http://biocc.hrbmu.edu.cn/CancerSEA/home.jsp) (17) was accessed to conduct correlation analysis. The signaling pathway scores of each single cell were obtained by GSVA enrichment analysis based on related gene sets in CancerSEA database. CancerSEA is a tool to comprehensively resolve distinct functional states of cancer cells at the single-cell level. GSE81383 is composed of single-cell RNA-seq data with 307 cells mapping heterogeneity in a patient-derived melanoma culture. Expression correlation between PRRT3-AS1 and significant pathway related key genes was analyzed by GEPIA2.

### Location of PRRT3-AS1 and construction of competitive endogenous RNA (ceRNA) network

PRRT3-AS1 location was explored using the lncLocator online tool (http://www.csbio.sjtu.edu.cn/bioinf/lncLocator/) (18) and the lncATLAS online tool (http://lncatlas.crg.eu/) (19). The lncLocator online tool is an ensemble classifier-based predictor predicting the lncRNA subcellular localizations. The LnCeVar online tool (http://www.bio-bigdata.net/LnCeVar/search_quick.jsp) (20) based on the TCGA-SKCM cohort was employed to predict the miRNA binding site of PRRT3-AS1 and the CeRNA network. LnCeVar is a comprehensive database that provides genomic variations that disturb lncRNA-associated competing endogenous RNA (ceRNA) network regulation curated from the published literature and high-throughput data sets.

### Methylation analysis of PRRT3-AS1

In the TCGA-SKCM cohort, the chromosome location of PRRT3-AS1 was revealed using the UCSC Genome Browser online tool (https://genome.ucsc.edu/), and the relationship between the expression of PRRT3-AS1 and the DNA methylation of PRRT3-AS1 was determined using the Xena online tool (https://xena.ucsc.edu/) (21). UCSC completed the first working draft of the human genome assembly and ensure free public access to the genome and the information it contains. Downloaded data from UCSC Xena were analyzed *via* the Scatterstats application in the Hiplot online tool (https://hiplot.com.cn/basic/ggscatterstats). OS was compared between the high-methylation group and the low-methylation group.

### Immune infiltration and immunotherapy response of PRRT3-AS1

Immune infiltration of PRRT3-AS1 in the TCGA-SKCM cohort was assessed by utilizing immunedeconv package. The ‘ggstatsplot’ package was used to draw the correlations between gene expression and immune score, the ‘pheatmap’ package was used to draw multi-gene correlation. RNA-sequencing expression (level 3) profiles and corresponding clinical information for PRRT3-AS1 were downloaded from the TCGA dataset. Potential ICB response was predicted with TIDE algorithm. TIDE uses a set of gene expression markers to evaluate two different mechanisms of tumor immune escape, including dysfunction of tumor-infiltrating cytotoxic T lymphocytes (CTLS) and rejection of CTLS by immunosuppressors. Patients with High TIDE score showed poor efficacy of immune checkpoint blockade therapy (ICB) and short survival after ICB (22). Used Spearman’s correlation analysis to describe the correlation between quantitative variables without a normal distribution. P values less than 0.05 were considered statistically significant (*P < 0.05).

## Results

### A 5-marker-lncRNAs model of significant prognostic value was constructed

**Figure 1:**
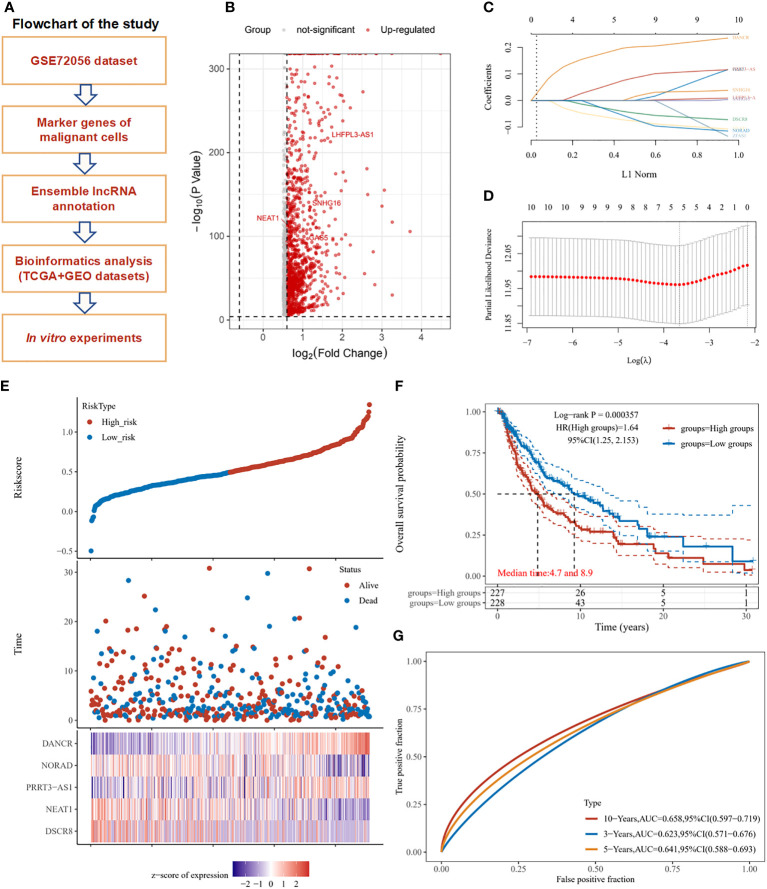
Study workflow and marker lncRNAs prognosis model construction in SKCM. **(A)** Workflow of the study. **(B)** Volcano plot exhibits the marker RNAs of melanoma cells in GSE72056. **(C)** LASSO coefficient profiles of the marker lncRNAs for overall survival in GSE72056. **(D)** Partial likelihood deviance of overall survival for the LASSO coefficient profiles. Five features with non-zero coefficients were selected by optimal lambda. **(E)** Melanoma patients were sorted by risk score, based on which patient status and the selected lncRNA expression of each patient was exhibited. Distribution of risk score (upper figure), OS (middle figure), and heat map of DANCR, NORAD, PRRT3-AS1, NEAT1, and DSCR8 expression in the GSE72056 dataset (lower figure). **(F)** Survival curves and patient status of 5-marker-lncRNAs. **(G)** ROC curves compare the prognostic accuracy of the classifier in melanoma patients using AUCs at 1, 5, and 10 years to assess prognostic accuracy.

The overall design and flowchart of this study are shown in **Figure 1A**. In **Figure 1B**, a volcano plot exhibits the distribution of the marker RNAs in SKCM cells. A total of 10 lncRNAs with the most prominent contributions were filtered (**Figure 1C**). LASSO Cox regression analysis showed that 5 lncRNAs model including DANCR, NORAD, PRRT3-AS1, NEAT1, and DSCR8 meet the best degree of fitting. (**Figure 1D**).

In the GSE72056 dataset, as the risk score increased, the patient’s mortality gradually increased, the expression levels of NORAD, NEAT1, and DSCR8 decreased, and the expression levels of DANCR and PRRT3-AS1 gradually increased (**Figure 1E**). According to the median risk score, the TCGA-SKCM cohort was divided into a high-risk group (n = 227) and a low-risk group (n = 228). The OS of the high-risk group was significantly lower than that of the low-risk group (*P* < 0.001). The median OS time of the high-risk group was 4.7 years, and that of the low-risk group was 8.9 years (**Figure 1F**). The receiver operating characteristic (ROC) curve analysis also showed the risk score of the 5-marker-lncRNAs model had a good predictive ability for OS at 1, 5, and 10 years in TCGA SKCM cohort. Training data was split by the median of the average expression of filtered factors. (**Figure 1G**). Neutrophil count was found to be significantly negatively correlated with the risk score of the model, whereas B cell, CD4+ T cell, CD8+ T cell, macrophage, and myeloid dendritic cell expression had no statistically significant association with risk score (**Supplementary Figure 1**).

### PRRT3-AS1 combining DANCR provided significant prognostic value in SKCM

**Figure 2:**
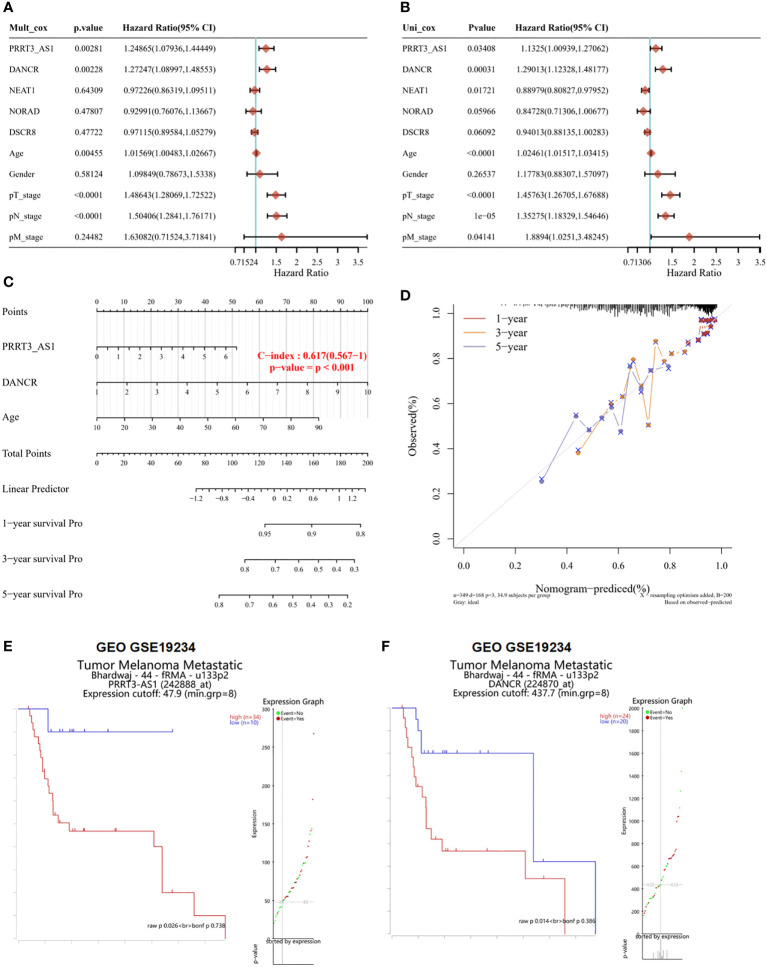
The identification of independent prognostic factors for OS and the development of the nomogram in TCGA-SKCM. Survival analysis of selected lncRNAs in the GEO dataset. Forest plot presenting the multivariate Cox regression analysis **(A)** and univariate Cox regression analysis **(B)** of selected lncRNAs. Nomogram **(C)** and the calibration curves **(D)** for predicting 1-, 3-, and 5-year overall survival of PRRT3-AS1 and DANCR. Overall survival curves and patient status according to the expression of PRRT3-AS1 **(E)** and DANCR **(F)**.

From multivariate Cox proportional hazard regression, PRRT3-AS1 (*P* = 0.00281, HR = 1.24865, 95% CI = 1.0736–1.44449), DANCR (*P* = 0.00228, HR = 1.27247, 95% CI = 1.08997–1.48553), and age (*P* = 0.00455, HR = 1.01569, 95% CI = 1.00483–1.02667) were identified as significant positive prognostic factors for pT-stage (*P* < 0.0001, HR = 1.48643, 95% CI = 1.28069–1.72522) and pN-stage (*P* < 0.0001, HR = 1.50406, 95% CI = 1.28410–1.76171) (**Figure 2A**). From univariate Cox proportional hazard regression, PRRT3-AS1 (*P* = 0.03408, HR = 1.1325, 95% CI = 1.00939–1.27062), DANCR (*P* = 0.00031, HR = 1.29013, 95% CI = 1.12328–1.48177), and age (*P* < 0.0001, HR = 102461, 95% CI = 1.01517–1.02415) were identified as significant positive prognostic factors for pT-stage (*P* < 0.0001, HR = 1.45763, 95% CI = 1.26705–1.67688), pN-stage (*P* = 1e-05, HR = 1.35275, 95% CI = 1.18329–1.54646), and pM-stage (*P* = 0.4141, HR = 1.88940, 95% CI = 1.0251–3.48245) as well as significant positive prognostic factors for OS (**Figure 2B**). A combination of marker lncRNAs PRRT3-AS1 and DANCR and the clinical feature age was selected as a prognostic model. Afterward, we developed a novel prognostic nomogram for SKCM patients based on the new model (PRRT-AS1, DANCR expression and age) with the c index of 0.617 (P < 0.001, 95% CI = 0.567–1) which means 61.7% Calibration of prediction the 1, 3, and 5-years OS in the SKCM patients (**Figure 2C**). Calibration curves of the nomogram are shown in **Figure 2D**.

According to the median risk score of PRRT3-AS1, the TCGA-SKCM cohort was divided into a high-risk group (n = 224) and a low-risk group (n = 225). The disease-free survival (DFS) of the high-risk group was significantly lower than that of the low-risk group (*P* = 0.0294). The median DFS time of the high-risk group was 5.5 years, and that of the low-risk group was 10.4 years. The ROC curve also showed that the DFS of the high-risk group was lower than that of the low-risk group (**Supplementary Figure 2A**). Similar results were found with DANCR, as the median DFS time of the high-risk group was 5.5 years and that of the low-risk group was 10.6 years, and the log-rank *P* value was 0.0109 (**Supplementary Figure 2B**).

The results of the survival analysis of GSE19234 also showed that patients with high expression of PRRT3-AS1 (high expression group samples = 34, low expression group samples =10, *P* = 0.026) (**Figure 2E**) and DANCR (high expression group samples = 24, low expression group samples = 20, P = 0.014) (**Figure 2F**) had a poorer prognosis than patients with low expression of PRRT3-AS1 and DANCR. The group was divided by significant differential expression of investigated factor. All evidence collected thus far indicated that we had constructed a risk model with two excellent lncRNAs for the prognosis of patients with SKCM, and PRRT3-AS1 and DANCR are independent prognostic factors of SKCM.

### PRRT3-AS1 was highly expressed in more advanced melanoma and the copy number variation of PRRT3-AS1 predicted poor prognosis

**Figure 3:**
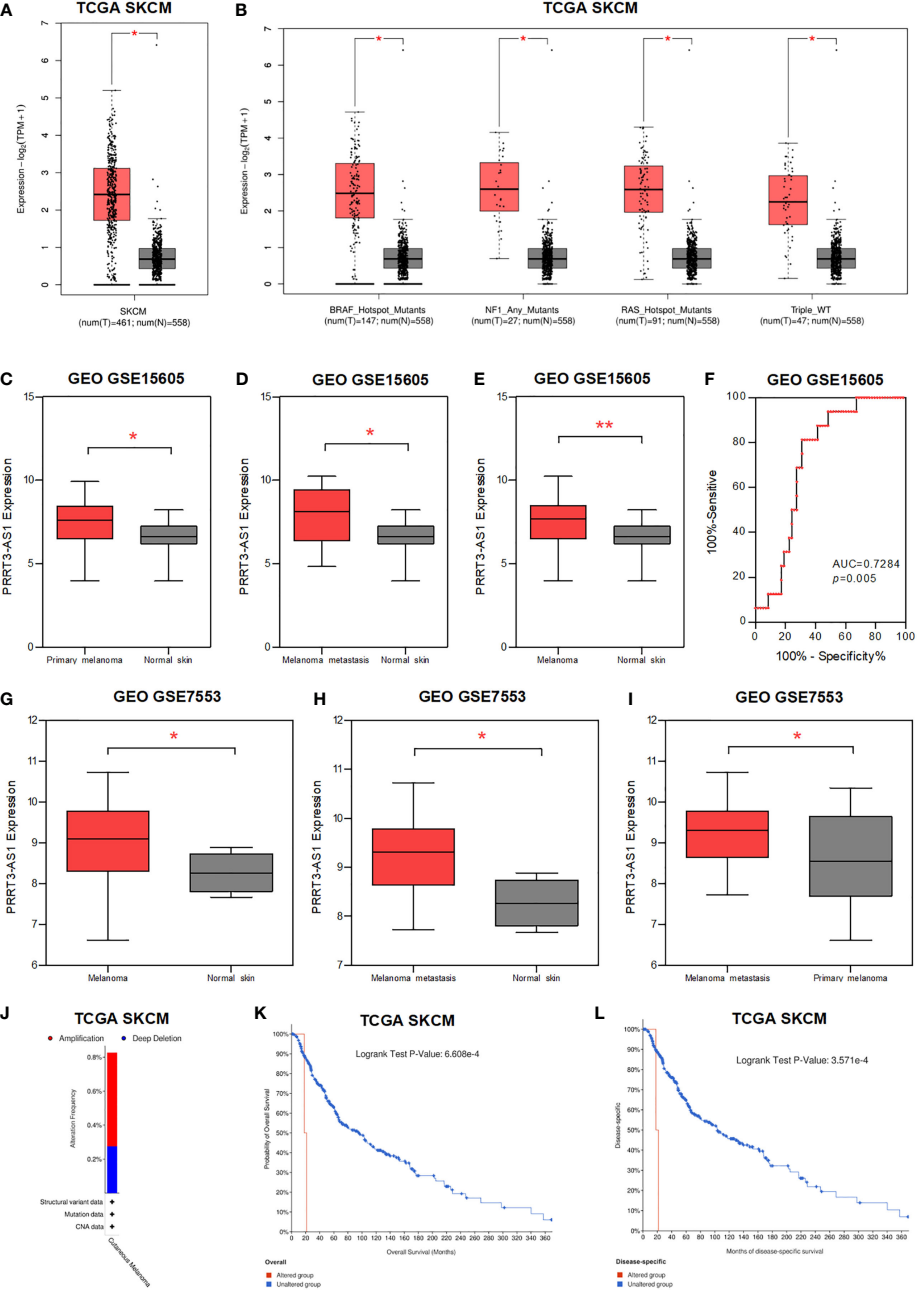
The expression and CNV of PRRT3-AS1 in SKCM. **(A)** The expression of PRRT3-AS1 in normal tissues and melanoma tissues in TCGA-SKCM. **(B)** The expression of PRRT3-AS1 in normal tissues and melanoma tissues with BRAF, NF1, RAS mutants, and Triple WT in TCGA-SKCM. The expression of PRRT3-AS1 in GSE15605, comparing normal skin tissues with primary melanoma **(C)**, metastatic melanoma **(D)**, and primary+metastatic melanoma **(E)** skin tissues. **(F)** ROC curves measuring the sensitive and specific values of PRRT3-AS1 in GSE15605. The expression of PRRT3-AS1 in GSE7553, comparing normal skin tissues with ordinary melanoma **(G)** and metastatic melanoma **(H)** skin tissues. **(I)** The expression of PRRT3-AS1 in metastatic melanoma and primary melanoma in GSE7553. **(J)** PRRT3-AS1 amplification condition in TCGA-SKCM. Survival curves of PRRT3-AS1 altered grouping in TCGA skin cutaneous melanoma patients with overall survival **(K)** and months of disease-specific survival **(L)** in TCGA-SKCM. ^*^ *P* < 0.05, ^**^ *P* < 0.01.

We found PRRT3-AS1 was significantly highly expressed in melanoma, BRAF, NF1, RAS mutants, and Triple WT tissues (**Figures 3A, B**), whereas DANCR showed limited expression difference (**Supplementary Figures 3A, B**) (15). In the GSE15605 dataset, PRRT3-AS1 was found to be significantly highly expressed in primary melanoma, primary + metastatic melanoma, and melanoma skin tissues in comparison to normal skin tissues (**Figures 3C-E**), whereas DANCR showed no significant difference in expression (**Supplementary Figures 3C–E**). Meanwhile, the AUC value of PRRT3-AS1 was 0.7284 (*P* = 0.005, **Figure 3F**). In the GSE7553 dataset, PRRT3-AS1 was found to be significantly highly expressed when comparing melanoma skin tissues to normal skin tissues, primary+metastatic melanoma skin tissues to normal skin tissues, and primary+metastatic melanoma skin tissues to primary melanoma skin tissues (**Figures 3G–I**), whereas DANCR showed no significant differences in any of the comparisons (**Supplementary Figure 3F**).

About 0.5% amplification and 0.3% deep deletion of PRRT3-AS1 are shown in **Figure 3J** (16). With PRRT3-AS1 altered grouping, OS analysis (*P* = 6.608e-4) and months of disease-specific survival analysis (*P* = 3.571e-4) were conducted, which indicated significantly poor prognosis associated with PRRT3-AS1 alteration in the TCGA-SKCM cohort (**Figures 3K, L**).

### PRRT3-AS1 may be required for cancer cell migration of SKCM cells

**Figure 4:**
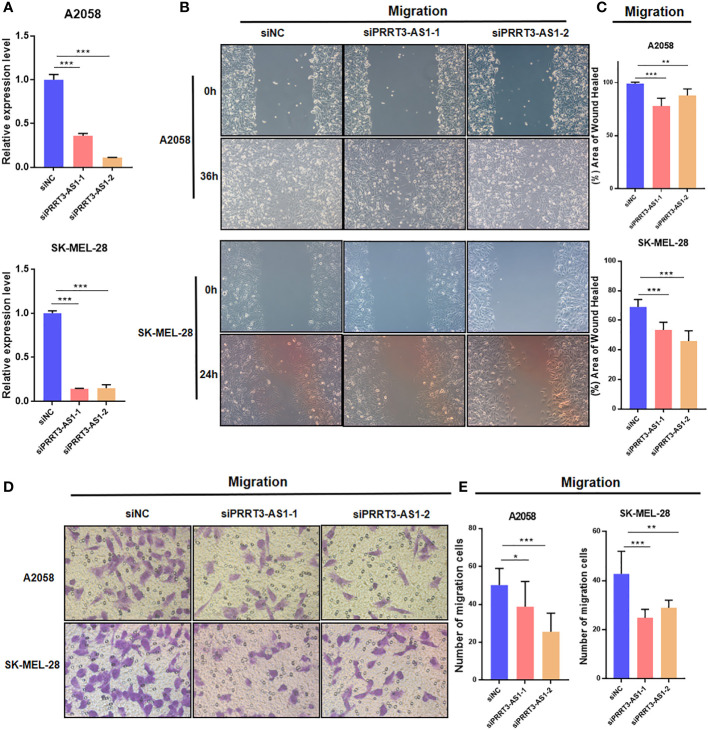
Cell model validation of PRRT3-AS1 in A2058 and SK-MEL-28 melanoma cells transfected with siPRRT3-AS1 and vector. **(A)** qPCR results of PRRT3-AS1 on RNA expression. Representative images **(B)** and quantitative analysis **(C)** of the results from the wound healing assay. Representative images **(D)** and quantitative analysis **(E)** of the results using transwell assay. ^*^ *P* < 0.05, ^**^ *P* < 0.01, ^***^ *P* < 0.001.

To identify the roles of PRRT3-AS1 in the development and progression of SKCM cells, we set up a PRRT3-AS1 downregulation model by siRNA transfection, after which we performed qPCR, wound healing, and transwell assays to explore the roles of PRRT3-AS1 in tumor migration in A2058 and SK-MEL-28 cell lines. The downregulation SKCM cell model was verified by qPCR (**Figure 4A**). In the wound healing assays, siRNA-induced downregulation of PRRT3-AS1 led to a decrease in the wound healing rate due to the significantly decreased cell migration ability in both the A2058 and SK-MEL-28 cell lines (**Figures 4B, C**). In the transwell assays, the decrease in PRRT3-AS1 expression gave rise to a significant decrease in the number of cells invading through the chamber in both the A2058 and SK-MEL-28 cell lines (**Figures 4D, E**). Taken together, the data indicated that PRRT3-AS1 may be required for cancer cell migration in SKCM cells.

### PRRT3-AS1 was related to epithelial-mesenchymal transition (EMT) signaling pathways

**Figure 5:**
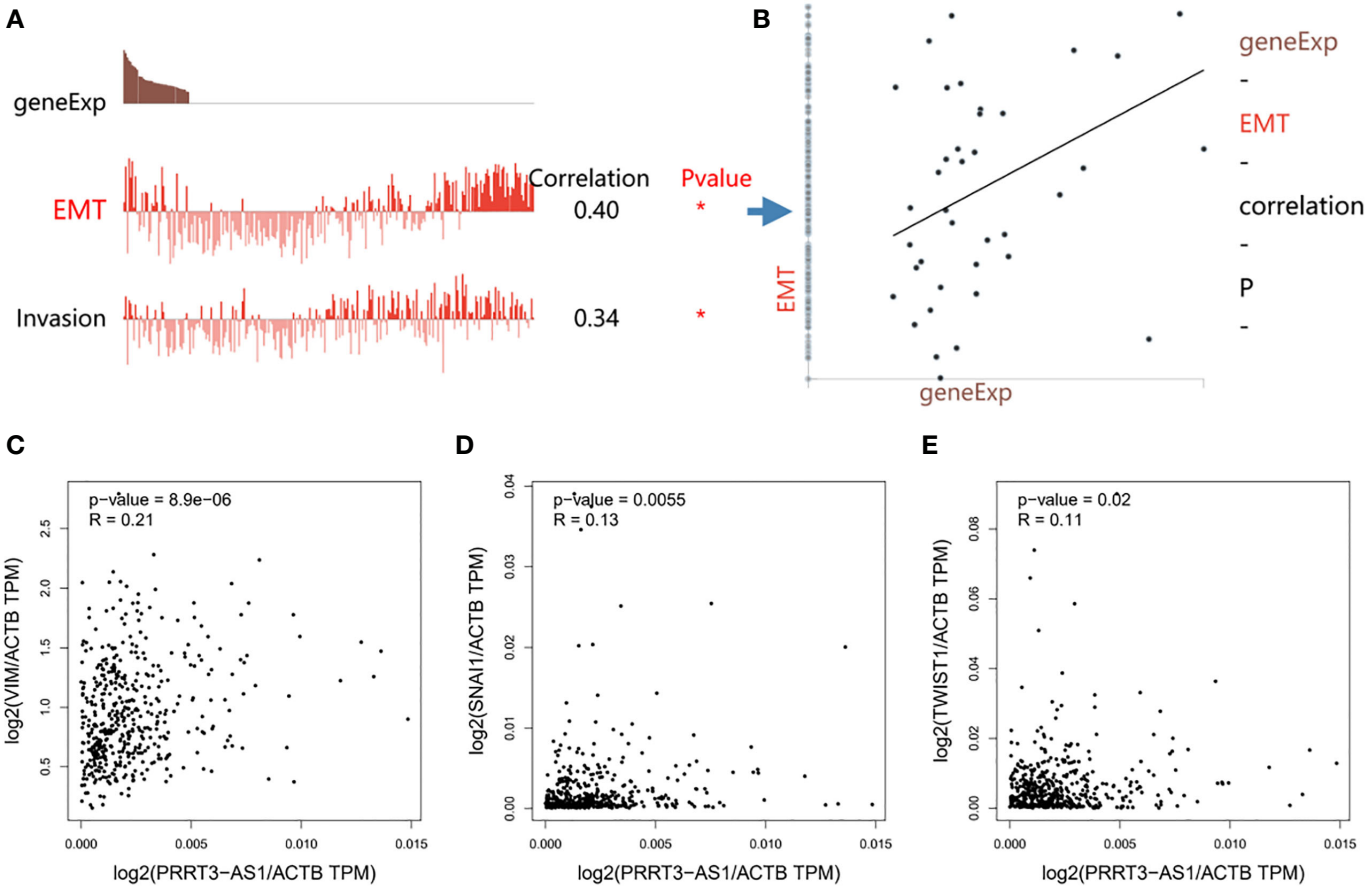
Functional relevance of PRRT3-AS1 in CancerSEA database. **(A)** Functional relevance of PRRT3-AS1 in melanoma. **(B)** Correlation between PRRT3-AS1 expression and EMT. The relation of PRRT3-AS1 expression and EMT-related genes VIM **(C)**, SNAI1 **(D)**, and TWIST1 **(E)**. *P < 0.05.

PRRT3-AS1 expression was significantly positively correlated with EMT (r = 0.40) and invasion (r = 0.34) signaling pathways in the GSE81383 dataset (**Figures 5A, B**) (17). Furthermore, by using GEPIA2, PRRT3-AS1 expression was found to be significantly positively related to the expression of EMT signaling genes VIM (*P* = 8.9e-06), SNAI1 (*P* = 0.0055), and TWIST1 (*P* = 0.02) in the TCGA-SKCM cohort (**Figures 5C-E**).

### PRRT3-AS1 was located in the cytoplasm and acted as an important signaling site in SKCM progression

The lncLocator illustrated that the cytoplasm contains most PRRT3-AS1 (**Supplementary Figure 5A**). Similarly, lncATLAS indicated that more PRRT3-AS1 is located in the cytoplasm than in the nucleus in most cell lines (**Supplementary Figure 5B**), allowing lncRNA to serve as an excellent miRNAs binding site in human cells.

Based on the TCGA-SKCM cohort, LnCeVar predicted that PRRT3-AS1 targets hsa-miR-328-3p (**Supplementary Figure 4C**) and that the CeRNA network of PRRT3-AS1 interacts with SFRP1, H2AFX, and CSNK2A2 (**Supplementary Figure 4D**). Furthermore, enrichment analysis showed that PRRT3-AS1 was involved in evading apoptosis, tissue invasion and metastasis, tumor-promoting inflammation, insensitivity to antigrowth signals, and so on, indicating PRRT3-AS1 is an important signaling site in SKCM progression (**Supplementary Figure 4E**).

### DNA methylation of PRRT3-AS1 was related to PRRT3-AS1 expression and the prognosis of SKCM

**Figure 6:**
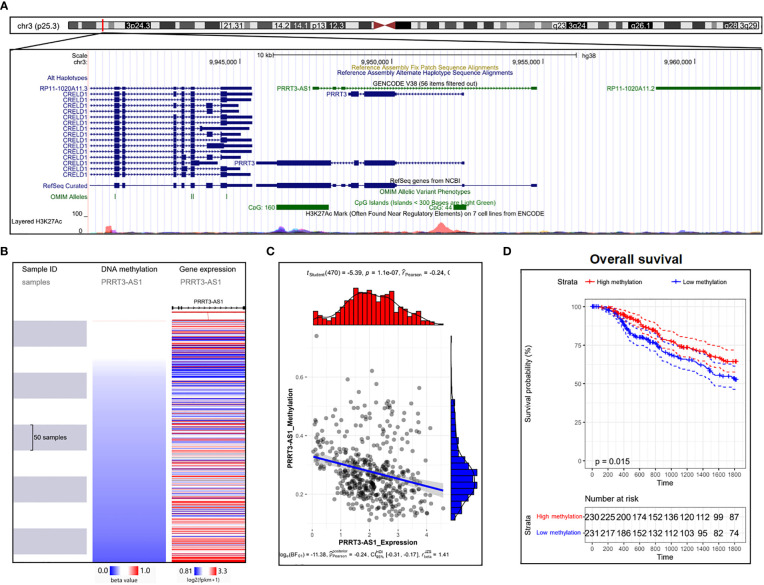
DNA methylation analysis of PRRT3-AS1 in TCGA-SKCM. **(A)** CpG islands of PRRT3-AS1 in the human genome. **(B)** Distribution of methylation and expression of PRRT3-AS1. **(C)** The distribution of methylation and the expression level of PRRT3-AS1. **(D)** Kaplan–Meier curve survival analysis between the high-methylation and low-methylation groups.

Two CpG islands were detected nearby, showing essential possibilities of DNA methylation (**Figure 6A**) (23). As the expression of PRRT3-AS1 increased, the DNA methylation of PRRT3-AS1 decreased (**Figure 6B**) (21) in the TCGA-SKCM cohort, and DNA methylation was significantly negatively related (Pearson’s Coefficient = −0.24) to PRRT3-AS1 expression (**Figure 6C**). In addition, the TCGA-SKCM cohort divided by median PRRT3-AS1 DNA methylation (high methylation group number = 230, low methylation group number = 231) showed the OS of the high-methylation group was significantly higher than that of the low-methylation group (**Figure 6D**).

### PRRT3-AS1 predicted immune infiltration and responses of immunotherapy

**Figure 7:**
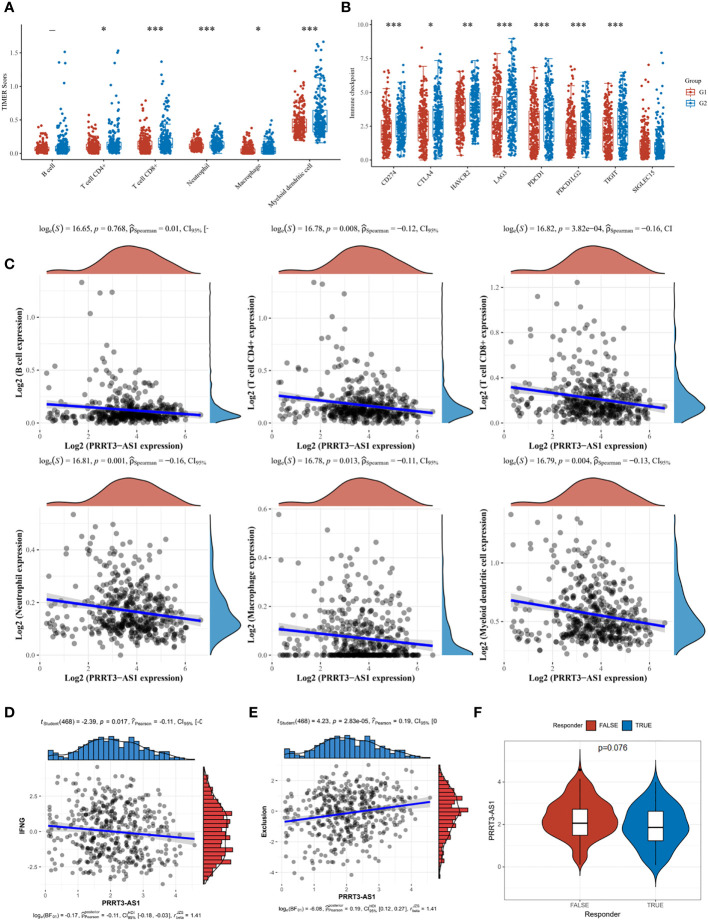
Immune cell infiltration landscape of PRRT3-AS1 in TCGA-SKCM. **(A)** Level of immune cell infiltration comparing PRRT3-AS1 high group and PRRT3-AS1 low group. **(B)** Level of immune checkpoint comparing PRRT3-AS1 high group and PRRT3-AS1 low group. **(C)** The distribution of B cell, CD4+ T cell, CD8+ T cell, neutrophil, macrophage, myeloid dendritic cell count, and the expression level of PRRT3-AS1. **(D)** The co-expression of interferon-gamma (IFNG) and PRRT3-AS1. **(E)** The co-expression of immune exclusion score and PRRT3-AS1. **(F)** PRRT3-AS1 expression in the immunotherapy response group and immunotherapy non-response group. ^*^ *P* < 0.05, ^**^ *P* < 0.01, ^***^ *P* < 0.001.

In the TCGA cohort, T cell CD4+ (*P* = 0.008, r = −0.12), T cell CD8+ (*P* = 3.82e-04, r = −0.16), neutrophil (*P* = 0.001, r = −0.16), macrophage (*P* = 0.013, r = −0.11), and myeloid dendritic cell (*P* = 0.004, r = −0.13) were significantly enriched in the low-expressed PRRT3-AS1 group (**Figures 7A, C**). A heatmap of the analysis is shown in **Supplementary Figure 5**. Similarly, patients with low-expressed scores had significantly elevated expression of immune checkpoints CD274, CTLA4, HAVCR2, LAG3, PDCD1, PDCD1LG2, and TIGIT (**Figure 7B**). Interferon-gamma (IFNG) aggregation was negatively related to PRRT3-AS1 expression (*P* = 0.017, r = −0.11) (**Figure 7D**), and exclusion of immune cells was positively related to PRRT3-AS1 expression (*P* = 2.83e-05, r = 0.19) (**Figure 7E**). Potential immunotherapy response was predicted with the TIDE algorithm, and PRRT3-AS1 was expressed more in non-response groups (**Figure 7F**).

## Discussion

ScRNA-seq has emerged as a splendid tool for the transcriptional classification of cell types in various cancers. Here, we screened key differentially expressed lncRNAs based on SKCM scRNA-seq data and verified the lncRNAs models *via* GEO SKCM datasets and the TCGA-SKCM cohort. An important lncRNA, PRRT3-AS1, was detected through this process. Similarly, Zhangxiang et al. identified PRRT3-AS1 as a potential biomarker of resistance to invasive treatment for breast carcinomas (24). Moreover, PRRT3-AS1 has also been reported as a part of prognostic models for glioblastoma (25), prostate cancer (26), hepatocellular carcinoma (27), and prostate cancer (28). These findings suggest that PRRT3-AS1 may be an oncogenic biomarker. Therefore, PRRT3-AS1 likely has significant clinical application value. However, previous studies have not explicitly illustrated the specific functions and mechanisms of PRRT3-AS1.

Subsequently, our results suggest that PRRT3-AS1 may be required for cancer cell migration in SKCM through cellular experiments for the first time, suggesting that PRRT3-AS1 is not only a potential biomarker but also a potential therapeutic target of SKCM. Given this, we performed research to explore the possible biological mechanism of PRRT3-AS1. Our bioinformatics analysis revealed that PRRT3-AS1 may be associated with EMT-related signaling pathways, and the ceRNA mechanism may play a key role in the process. Meanwhile, Drug response analysis from Zhao Z revealed that PRRT3-AS1 is a potential resistance biomarker for paclitaxelin BRCA treatment in breast carcinomas (24). Further, PRRT3-AS1 and AL031985.3 were identified as immune-related prognostic lncRNAs in HCC patients according to study from Liang R (25). Zhang P found that Five lncRNAs including PRRT3-AS1 were found related to focal adhesion, extracellular matrix receptor interaction, and mitogen-activated protein kinase signaling pathways in prostate cancer (26). Fan L also revealed silencing of lncRNA PRRT3-AS1 can upregulate apoptosis and autophagy downregulate migration and invasion of PC cells through the mTOR signaling pathway (28). Through univariate and multivariate Cox regression analyses of the 7 lncRNAs signature, Yang S found that this risk score has good survival prediction effciency in HCC patients (27). These results suggest functions and mechanisms of PRRT3-AS1 may be heterogeneous in different types of cancer, and our study proposes a new mechanism of PRRT3-AS1.

Immunotherapy plays an important role in SKCM treatment today, and blocking antibodies to CTLA4 and PD1/PDL1 has improved survival rates for many patients. Front-line treatments, including ipilimumab with nivolumab or either nivolumab or pembrolizumab alone, have improved prognosis, and there are currently ongoing clinical trials involving atezolizumab and avelumab (29, 30). Yu et al. identified lncRNA as a useful biomarker for cancer immunotherapy based on the TCGA-SKCM cohort (31). However, effective biomarkers of SKCM immunotherapy are still needed because of tumor heterogeneity and immunological tolerance. Our study showed that PRRT3-AS1 was negatively correlated with the immune cell count and the expression of immune checkpoint genes. This suggested that PRRT3-AS1 may play an important role in suppressing the immune system in SKCM patients, and further analysis showed PRRT3-AS1 was also negatively correlated with immunotherapy response and immune effect responders. These results suggested PRRT3-AS1 may be a marker of immunotherapy response in SKCM, but this remains to be verified by a cohort study with a larger population. In summary, our study is significant because we discovered PRRT3-AS1 has the potential to serve as an immunotherapy biomarker in SKCM, meaning it has great potential value in clinical application.

Recent studies revealed that N6-methyladenosine (a very common modification in mRNA and DNA) silencing and methylation state are critical to the function of lncRNAs (32, 33). Therefore, we also explored the up-regulation mechanism of PRRT3-AS1 expression and found that the expression of PRRT3-AS1 in SKCM was significantly negatively correlated with its methylation level. This suggests that the overexpression of PRRT3-AS1 may be related to DNA methylation, but further study is needed to validate this finding in other types of malignancies.

However, there were two limitations in our study. First, the SKCM data in this paper were from European and American populations, and it remains unclear whether the conclusions apply to other populations, especially Asian populations. Second, the underlying molecular mechanism of PRRT3-AS1 still needs to be further explored.

## Conclusion

In this study, we discovered PRRT3-AS1 is a diagnostic, prognostic, and immunotherapy biomarker of SKCM. PRRT3-AS1 plays an important role in SKCM promotion, and its potential as a therapeutic target for SKCM warrants further study.

## Data availability statement

The raw data supporting the conclusions of this article will be made available by the authors, without undue reservation.

## Author contributions

ST and WZ designed the study. ST reviewed the manuscript. WZ, XX, ZH, and XZ performed cell experiments and bioinformatic analysis. WZ wrote the manuscript. YL, K-LC, JZ, and ST are the guarantors of this work and, as such, have full access to all data in the study and take responsibility for the integrity of the data and the accuracy of the data analysis. All authors approved the manuscript.

## Funding

This study was funded by the National Natural Science Foundation of China (82071101, 82002068), Guangdong Medical Research Foundation Project (A2020099, A2020538), Shantou Science and Technology Project ([2019]10602), 2020 Li Ka Shing Foundation Cross-Disciplinary Research Grant (2020LKSFG18B, 2020LKSFG02E), Guangdong University Innovation Team Project(2021KCXTD047), and Guangdong Science and Technology Special Fund (200114165897946, 210714106901245).

## Conflict of interest

The authors declare that the research was conducted in the absence of any commercial or financial relationships that could be construed as a potential conflict of interest.

## Publisher’s note

All claims expressed in this article are solely those of the authors and do not necessarily represent those of their affiliated organizations, or those of the publisher, the editors and the reviewers. Any product that may be evaluated in this article, or claim that may be made by its manufacturer, is not guaranteed or endorsed by the publisher.
