# Supplementary material for: The integration of single-cell sequencing, TCGA, and GEO data analysis revealed that PRRT3-AS1 is a biomarker and therapeutic target of SKCM
Source: Front Immunol. 2022 Sep 23;13:919145. doi: 10.3389/fimmu.2022.919145 (PMC9539251; doi:10.3389/fimmu.2022.919145)
Supplement: Supplementary file 1 [file DataSheet_1.zip › 919145_SupMaterial/Supplemental Table 1.docx]

Supplemental Table 1 Baseline tables for datasets

| Dataset | Source | sample scale | Expression Profile |
| --- | --- | --- | --- |
| GSE72056 | Tirosh I. Science. 2016 | 4645 cells, 19 patients | Single cell RNA-seq |
| GSE19234 | Bogunovic D. Proc Natl Acad Sci U S A. 2009 | 44 samples, 38 patients | Expression profiling by array |
| GSE15605 | Raskin L. J Invest Dermatol. 2009 | 74 samples | Expression profiling by array |
| GSE7553 | Riker AI. BMC Med Genomics. 2008 | 87 samples | Expression profiling by array |
| GSE81383 | GerberT. Oncotarget. 2017 | 307 cells, 3patients | Single cell RNA-seq |
| TCGA-SKCM |  | 477 samples, 470patients | Multiply molecular profile |
