# Supplementary material for: The integration of single-cell sequencing, TCGA, and GEO data analysis revealed that PRRT3-AS1 is a biomarker and therapeutic target of SKCM
Source: Front Immunol. 2022 Sep 23;13:919145. doi: 10.3389/fimmu.2022.919145 (PMC9539251; doi:10.3389/fimmu.2022.919145)
Supplement: Supplementary file 1 [file DataSheet_1.zip › 919145_SupMaterial/Supplemental Table 2.docx]

Supplemental Table 2 siRNA sequences used in this study

| Name | Sequence |
| --- | --- |
| siNC sense | UUCUCCGAACGUGUCACGUTT |
| siNC antisense | ACGUGACUCGUUCGGAGAATT |
| PRRT3-AS1 si-1 sense | UCUCCUUUCCAGGAUUCUACUTT |
| PRRT3-AS1 si-1 antisense | AGUAGAAUCCUGGAAAGGAGATT |
| PRRT3-AS1 si-2 sense | CGGCCUCACACUGAAUUAAUGTT |
| PRRT3-AS1 si-2 antisense | CAUUAAUUCAGUGUGAGGCCGTT |
