# Supplementary figures and images for: The integration of single-cell sequencing, TCGA, and GEO data analysis revealed that PRRT3-AS1 is a biomarker and therapeutic target of SKCM
Source: Front Immunol. 2022 Sep 23;13:919145. doi: 10.3389/fimmu.2022.919145 (PMC9539251; doi:10.3389/fimmu.2022.919145)

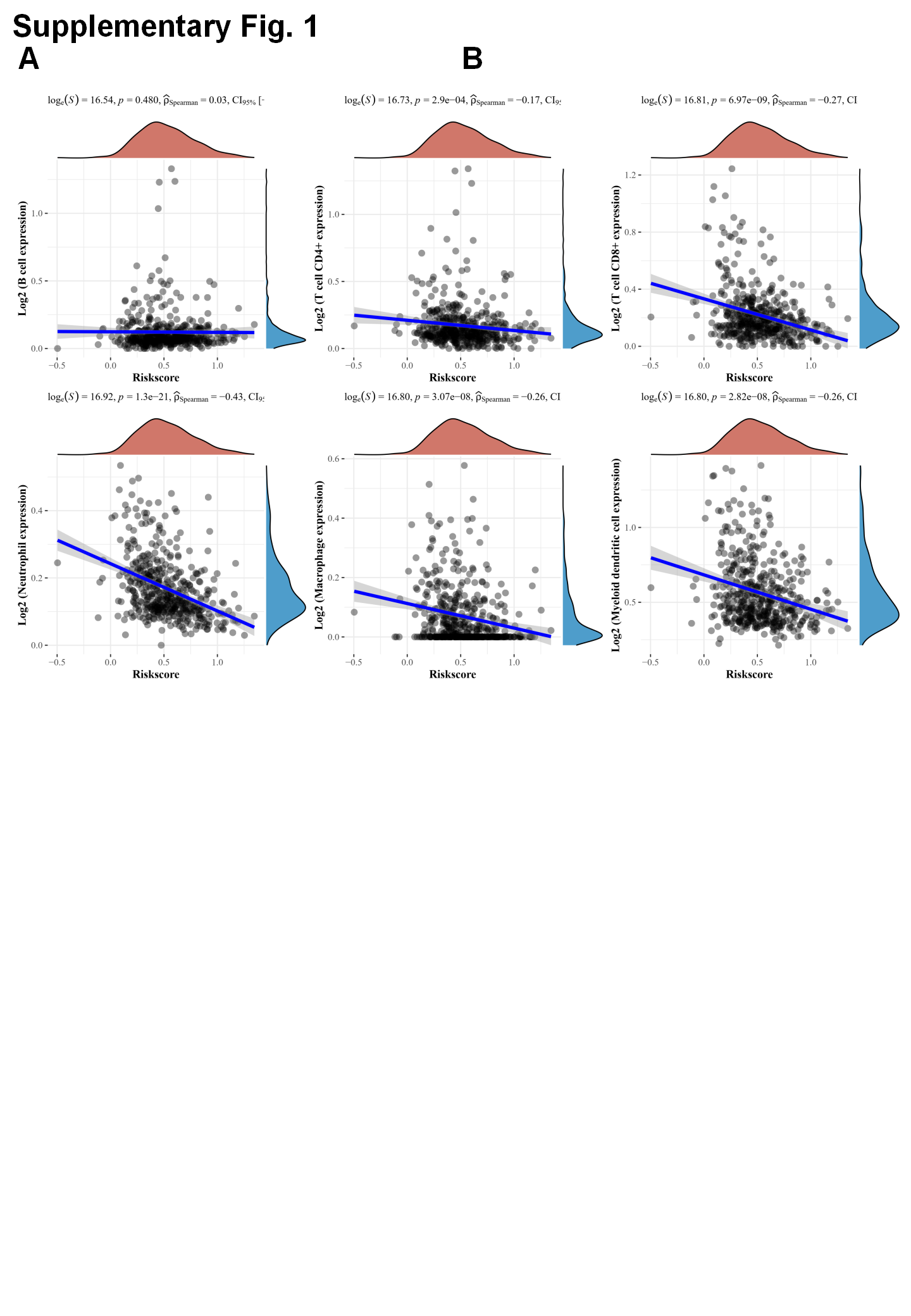

Supplement: Supplementary file 1 [file DataSheet_1.zip › 919145_SupMaterial/Supplemental Figure 1.TIF]

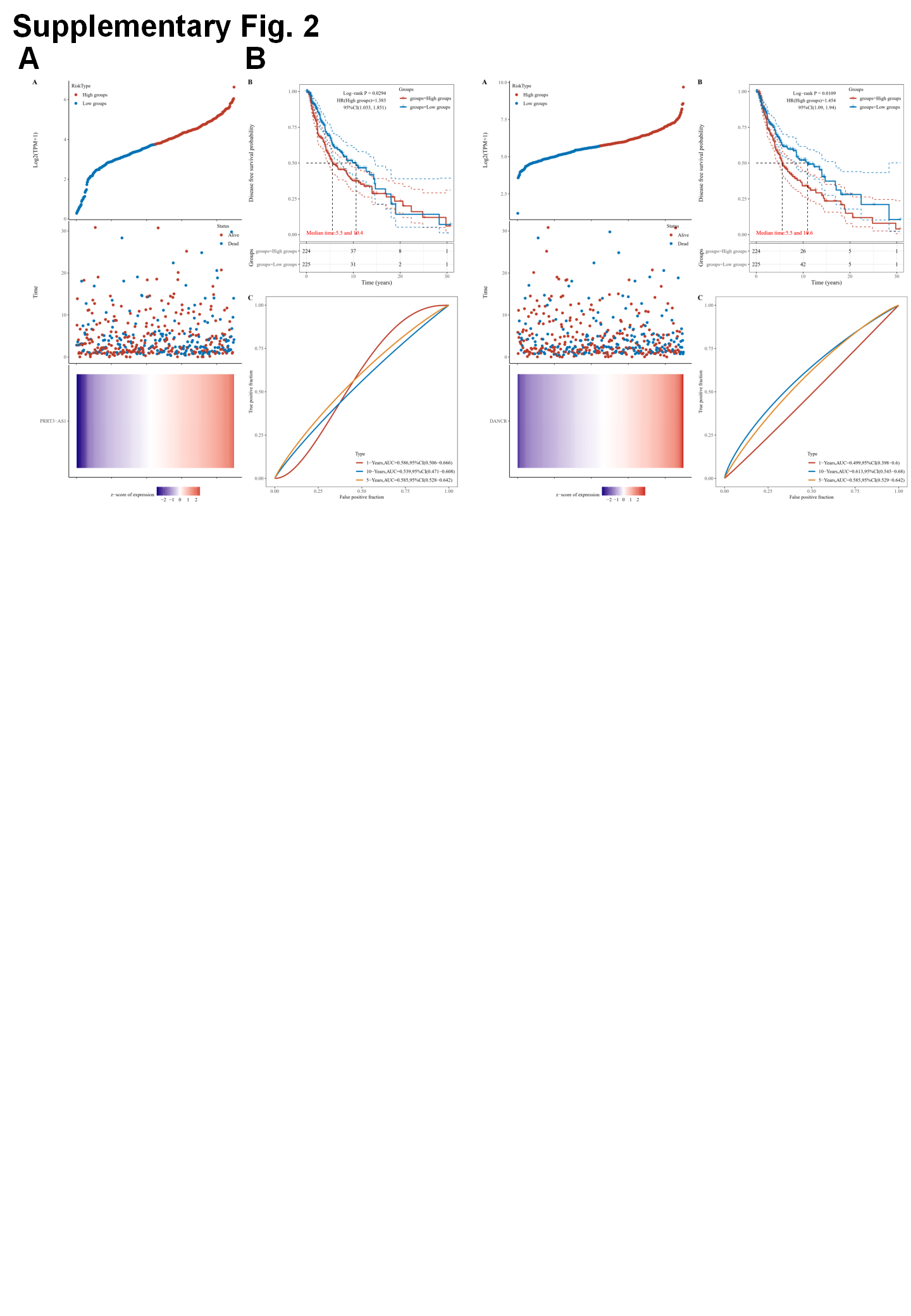

Supplement: Supplementary file 1 [file DataSheet_1.zip › 919145_SupMaterial/Supplemental Figure 2.TIF]

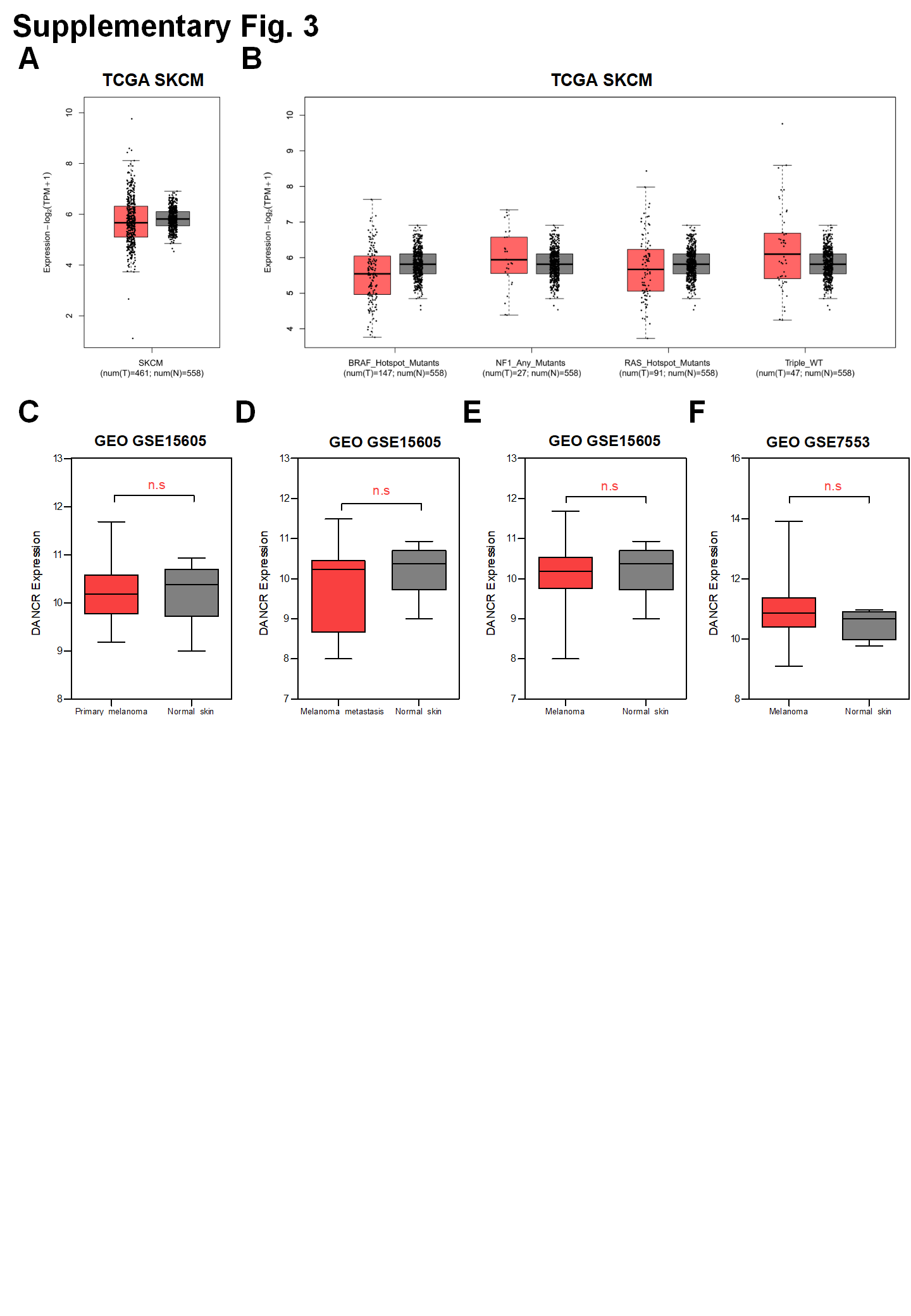

Supplement: Supplementary file 1 [file DataSheet_1.zip › 919145_SupMaterial/Supplemental Figure 3.TIF]

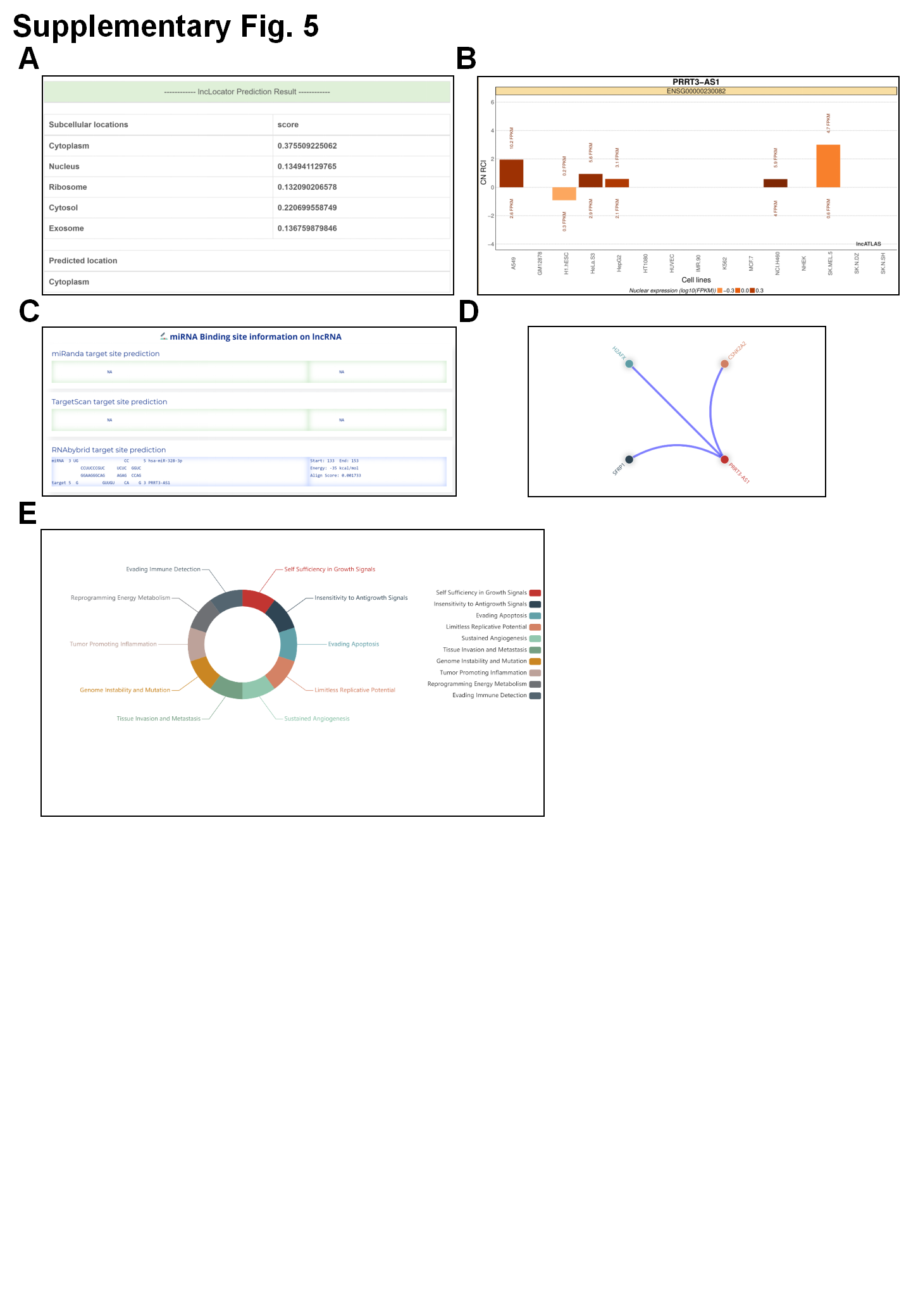

Supplement: Supplementary file 1 [file DataSheet_1.zip › 919145_SupMaterial/Supplemental Figure 4.TIF]

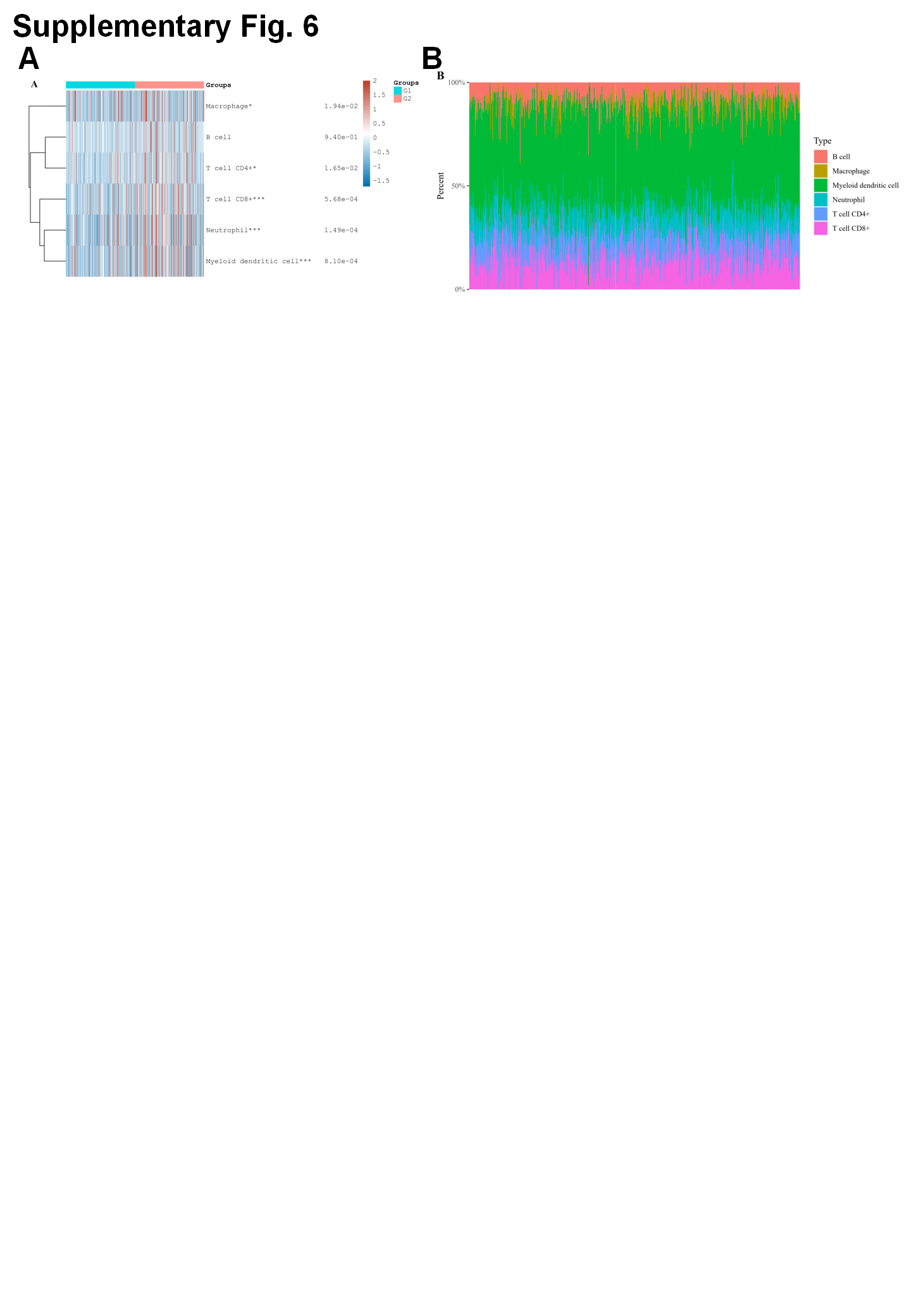

Supplement: Supplementary file 1 [file DataSheet_1.zip › 919145_SupMaterial/Supplemental Figure 5.TIF]
